# Identifying risk profile for adolescent e-cigarette use: A sex-stratified machine learning analysis

**DOI:** 10.1016/j.dadr.2026.100427

**Published:** 2026-03-10

**Authors:** Dae-Hee Han, Danyi Li, Raina D. Pang, Jimi Huh, Ming Li, Jessica L. Barrington-Trimis, Adam M. Leventhal

**Affiliations:** a Department of Behavioral, Social, and Health Education Sciences, Emory University, Atlanta, GA, USA; b Department of Health Policy and Management, Emory University, Atlanta, GA, USA; c Winship Cancer Institute of Emory University, Atlanta, GA, USA; d Department of Population and Public Health Sciences, University of Southern California, Los Angeles, CA, USA; e Institute for Addiction Science, University of Southern California, Los Angeles, CA, USA

**Keywords:** E-cigarettes, Risk Profile, Sex Difference, Machine Learning, Adolescents

## Abstract

**Introduction:**

Recent studies show that young females now report higher e-cigarette use than males, reversing prior trends. While sex differences in use are documented, little is known about underlying risk profiles. This study applied a machine learning (ML) approach to identify and compare predictors of adolescent e-cigarette use by sex.

**Methods:**

We analyzed cross-sectional data from 1829 9th graders in Southern California (M=14.6 years; 54.7% female) surveyed in 2024. Gradient Boosting Machine, an ML algorithm well-suited for binary classification tasks, was employed to develop past 30-day e-cigarette use prediction models by sex. We additionally fitted a model that combined both females and males to assess overall risk factors. Sixty-eight self-reported variables across conceptual domains were included, and the top 10 predictors per model were identified using scaled importance scores.

**Results:**

Overall, 3.6% (n = 66; 3.7% females, 3.5% males) reported past 30-day e-cigarette use. In the female model, depression and post-traumatic stress disorders emerged as leading predictors, but not for males. Top risk factors in the male model included beliefs about and susceptibility to e-cigarette and cannabis use. In the combined model, the strongest predictors were primarily cannabis use and peer e-cigarette use. Model performance was moderate, with area under the receiver operating characteristic curve values of 0.86–0.88 and area under the precision-recall curve values of 0.19–0.54.

**Conclusions:**

The findings of this study underscore the importance of considering sex differences when identifying risk profiles associated with e-cigarette use and developing targeted prevention and intervention programs for adolescents.

## Highlights

• Machine learning identified sex-specific risk profiles for youth e-cigarette use.
• Cannabis use was the strongest predictor of e-cigarette use across sexes.
• Mental health symptoms predicted e-cigarette use only among female adolescents.
• Prevention integrating mental health and e-cigarette use is needed for female youth.

## 1 Introduction

Over the past decade, e-cigarette use among adolescents increased substantially in many parts of the world. Surveillance reports from multiple countries indicate that this trend persists even in countries with robust tobacco control frameworks, underscoring the products’ accessibility and appeal to adolescents (World Health Organization, 2025). In the United States, despite recent declines in prevalence (Han et al., 2025), e-cigarettes remained most widely used nicotine product among adolescents since 2014 (Arrazola et al., 2015, Jamal et al., 2024). The National Youth Tobacco Survey (NYTS) reported that in 2024, approximately 6% of US high and middle school students (1.63 million youth) reported current (past 30-day) use of e-cigarettes (Arrazola et al., 2015, Arrazola et al., 2015). E-cigarette use among adolescents poses potential health concerns, including the development of nicotine dependence that may interfere with brain development (Castro et al., 2023) and adverse mental health outcomes (Javed et al., 2022, Leventhal et al., 2016).

Prior research has documented sex differences in e-cigarette use prevalence and patterns in young populations (Kong et al., 2017, Pang et al., 2020, Seidenberg et al., 2024). A 2022 systematic review of socio-ecological correlates of e-cigarette use among adolescents and young adults showed that among 33 studies on sex/gender differences, 20 reported that being male is a “risk factor” for e-cigarette use, while only 2 reported it as a protective factor (Han and Son, 2022). Another review study of adolescents revealed that males generally report higher e-cigarette use prevalence (Kong et al., 2017). However, nationally representative surveys of adolescents, such as the NYTS, the Youth Risk Behavior Surveillance System (Seidenberg et al., 2024), and the Monitoring the Future study (Han et al., 2025), have recently documented a reversed trend, with females reporting higher e-cigarette use prevalence than males. This reversal may be partly attributable to the increase in flavored e-cigarette use (Centers for Disease Control and Prevention, 2024), which is more prevalent among females (DeVito et al., 2025), and may help explain the recent trend. These shifting trends highlight the need to move beyond investigating prevalence differences and toward understanding the multifaceted factors that may differentially shape e-cigarette use among males and females. However, no prior research has comprehensively explored or compared the potential risk factors for e-cigarette use by sex – an approach that requires modern analytical techniques capable of handling high-dimensional data patterns.

Mental health and behavioral factors have also been identified as key risk factors for adolescent e-cigarette use. For example, mental health symptoms, including depression, panic disorder, and anhedonia, have been positively associated with e-cigarette use (Leventhal et al., 2016). Similarly, a national longitudinal study of U.S. adolescents found that higher levels of internalizing and externalizing symptoms were associated with an increased risk of e-cigarette initiation (Buu et al., 2021). In addition, substance use behaviors, particularly cannabis use, have also been identified as important predictors of e-cigarette use. For instance, any form of cannabis product use (e.g., smoking, vaping, hemp/cannabidiol) has been associated with increased likelihood of persistent e-cigarette use during adolescence (Han et al., 2024). However, much of the existing evidence on risk factors for adolescent e-cigarette use has relied primarily on regression-based modeling approaches.

Machine learning (ML) algorithms are powerful tools for building prediction models in a data-driven way, considering multiple potential predictors (Sarker, 2021). Gradient Boosting Machine (GBM) is a powerful supervised ML algorithm well-suited for binary classification while addressing some of the limitations of conventional logistic regression models (Natekin and Knoll, 2013). Although e-cigarette use is a common risk behavior among adolescents, data on substance use behaviors are often highly skewed (i.e., rare outcome), leading to increased risk of Type II errors when using logistic regression (Han et al., 2020, Japkowicz and Stephen, 2002). Another advantage of ML methods is their ability to avoid the challenges of multicollinearity and overfitting that conventional statistical approaches often encounter when incorporating a large number of predictor variables (Midi et al., 2010, Morgenstern et al., 2021). For these reasons, a growing body of research has adopted ML methods in tobacco research, leveraging survey data (Fu et al., 2023). For example, two recent applied ML studies have identified risk factors for adolescent e-cigarette use using the Population Assessment of Tobacco and Health (PATH) Study. Han et al., analyzing PATH data from 2013 to 2018 with penalized logistic regression, identified social media use as an emerging and significant predictor of current e-cigarette use (Han et al., 2021). Le et al. used 2017–2019 PATH data and applied a combination of ML algorithms to identify key risk factors for e-cigarette initiation, highlighting the important role of familial and peer relationships in shaping initiation behaviors (Le, 2023). While these studies leveraged nationally representative data, their ability to identify nuanced risk factors was limited by the fixed set of survey items available in those datasets (Han et al., 2021). This highlights the need for more adaptable and contemporary data sources that capture a broader and evolving spectrum of social-behavioral determinants.

The current study sought to develop supervised ML models predicting adolescent current e-cigarette use by sex, utilizing data from Southern California. This analytical approach was employed to build separate prediction models for male and female groups, enabling the identification of predictors that may be uniquely relevant to each group. This study was exploratory in nature and explored sex-based differences in the risk profiles for current e-cigarette use.

## 2 Methods

### 2.1 Data and study sample

Data were obtained from the Epidemiology and Public Health Investigation of Youth in California (EPIC) Study, an ongoing survey that began in Spring 2024, administered twice annually, with a cohort of ninth-grade students recruited from eight high schools in Southern California. Data are collected via electronic surveys administered during class time on substance use (e.g., nicotine, cannabis, alcohol) as well as mental wellness, social media engagement, and other relevant health behaviors. Between February and June 2024, a total of 1854 students completed the spring 2024 survey, constituting the first wave of the EPIC Study. For the current analysis, the sample was restricted to 1829 students with complete data on the study outcome (e-cigarette use) and the stratification variable (sex at birth).

#### 2.1.1 Ethics statement

Written parental consent and student assent were obtained prior to data collection. This study was approved by the University of Southern California Institutional Review Board.

### 2.2 Measures

*Outcome variable*. Participants reported the number of days that they used e-cigarettes in the past 30 days. Responses were recoded into a dichotomous outcome variable: past 30-day e-cigarette use (yes/no).

*Predictor variables*. This study incorporated a comprehensive set of predictor variables organized into distinct conceptual domains, selected from the full range of measures available in the EPIC Study and informed by established associations with e-cigarette use documented in the literature (Barrington-Trimis et al., 2015, Leventhal et al., 2015, Leventhal and Zvolensky, 2015). Specifically, included domains were: sociodemographic characteristics (e.g., age, race/ethnicity); past 30-day substance use behaviors (e.g., combustible tobacco, cannabis); e-cigarette susceptibility and belief measures; self-reported psychiatric symptoms (e.g., children’s anxiety and depression scale [RCADS]); perceived stress scale (PSS), impulse behavior scale (UPPS-P), biggest issues that teens face, phone use behaviors (e.g., frequency of social media use, multidimensional Facebook intensity scale [MFIS]); perceived neighborhood disorder, physical and mental health diagnoses made by a health professional (e.g., asthma, diabetes, attention-deficit/hyperactivity disorder, depression, PTSD); parent–adolescent communication scale (RPAC); and everyday discrimination scale (EDS). In total, 68 predictor variables were included in the analysis (see **Appendix Table A.1**).

### 2.3 Model development

To identify and compare key predictors that may differ between male and female participants, separate ML models were developed for males and females. Specifically, we developed a supervised ML model, where a set of candidate predictors was used to predict e-cigarette use through learned mapping functions. The dataset was randomly divided into a training set (70%) and a test set (30%), providing adequate data for training (model development) while preserving a sufficiently large holdout set for performance evaluation. To tune model hyperparameters, we implemented 5-fold cross-validation within the training data. This approach optimized key hyperparameters (e.g., the maximum depth of individual decision trees and the total number of trees constructed within the model). Grid search was used to identify the optimal combination of hyperparameter values during cross-validation. Specific search ranges and selected values are detailed in **Appendix Table A.2**. Once tuning was complete, final predictive models for e-cigarette use were fitted using the optimal hyperparameters. The process of ML model construction and hyperparameter tuning followed methodologies consistent with prior work (Han et al., 2020, Suchting et al., 2018). We additionally fitted a model that combined both females and males to assess overall risk factors. To assess the direction of associations between top predictors and e-cigarette use, we fitted binary logistic regression models using the combined male and female sample.

### 2.4 Statistical analysis

In line with prior studies (Han et al., 2021, Han et al., 2020), we evaluated the performance of ML models using two metrics: (1) the area under the receiver operating characteristic curve (AUROC) and (2) the area under the precision-recall curve (AUPRC). The AUROC measures a model’s ability to discriminate between individuals with and without the outcome of interest by plotting true positive rates against false positive rates across all possible classification thresholds (Bradley, 1997). In contrast, the AUPRC assesses a model’s ability to correctly identify positive cases by charting precision (i.e., positive predictive value) against recall (i.e., sensitivity or true positive rate, Davis, Goadrich, 2006). These two metrics are commonly employed to evaluate the discriminatory ability of predictive models in studies of substance use behaviors (Han et al., 2021, Han et al., 2020, Oh et al., 2023, Park et al., 2021). AUROC of 0.7–0.8 is generally considered acceptable, 0.8–0.9 good, and ≥0.9 excellent discrimination (White et al., 2023). Unlike AUROC, AUPRC does not have absolute, universally accepted interpretive thresholds and must be interpreted in relation to outcome prevalence and application context. AUPRC is increasingly recognized as an appropriate primary metric for evaluating model performance in imbalanced classification contexts (Saito and Rehmsmeier, 2015).

Following model evaluation using these metrics, we further examined the relative importance of predictive variables, which is measured by a predictor’s selection frequency in tree building and its contribution to reducing overall squared error (Bradley, 1997). When a node splits on a feature, its importance is the decrease in squared error from the parent to child nodes, reflecting the reduction in response variance. Aligned with prior applied ML research (Han et al., 2021, Han et al., 2020), this study identified and reported the top 10 predictors with the highest predictive importance from the GBM models. This was done separately for each model. Missing data for predictor variables (sociodemographic factors: ~23.2%; e-cigarette susceptibility/belief: ~12.3%; psychiatric symptoms: 13.3%, phone use behaviors: 10.8%, physical and mental health diagnoses made by a health professional: 11.5%; everyday discrimination scale: 13.3%, predictors not listed had less than 10% missingness) were imputed using multiple imputation by chained equations, generating five imputed datasets with 40 iterations conducted for each (White et al., 2011). All analyses were performed using R version 4.4.3 and the H2O R interface (version 3.44.0.3), an open-source, Java-based platform that provides a broad suite of machine learning and big data tools. Additional information on the algorithms implemented in H2O can be found in its documentation (H2O.ai, 2025).

## 3 Results

Table 1 displays participant characteristics stratified by sex at birth for a sample of 1829 adolescents. No significant sex differences were observed across sociodemographic characteristics except for sexual minority identity. Overall, 3.6% reported past 30-day e-cigarette use, with similar prevalence among females (3.7%) and males (3.5%) – there was no statistically significant difference in e-cigarette use by sex.

**Table 1:** Participant characteristics.

**Variables**	**Overall sample** **(n = 1829)**	**Sex**^**a**^		
		**Female (n = 1001)**	**Male (n = 828)**	***p*****-value**
**Past 30-day e-cigarette use**	66 (3.6)	37 (3.7)	29 (3.5)	.92
**Demographics**				
Age, M (SD)	14.6 (0.5)	14.6 (0.5)	14.6 (0.5)	.44
Sexual minority identity^b^	335 (18.3)	256 (25.6)	79 (9.5)	<.01
Race/ethnicity				.16
Hispanic or Latino	886 (48.4)	494 (49.4)	392 (47.3)	
Non-Hispanic Asian	485 (26.5)	262 (26.2)	223 (26.9)	
Non-Hispanic Black	44 (2.4)	27 (2.7)	17 (2.1)	
Non-Hispanic White	230 (12.6)	109 (10.9)	121 (14.6)	
Non-Hispanic multi-racial or multi-ethnic	126 (6.9)	76 (7.6)	50 (6.0)	
Non-Hispanic another race/ethnicity^c^	58 (3.2)	33 (3.3)	25 (3.0)	
Parents attended college	935 (51.1)	503 (50.2)	432 (52.2)	.44

*Note.* Results are n (%) unless otherwise specified.

^a^ Sex at birth. Participants who did not disclose their sex (n = 17) or did not provide a response (n = 8) were excluded from the study.

^b^ Asexual, bisexual, gay, lesbian, pansexual, queer, questioning/unsure, or another non-heterosexual identity.

^c^ American Indian/Alaska Native, Black or African American, or Native Hawaiian/Pacific Islander.

**Fig. 1:**
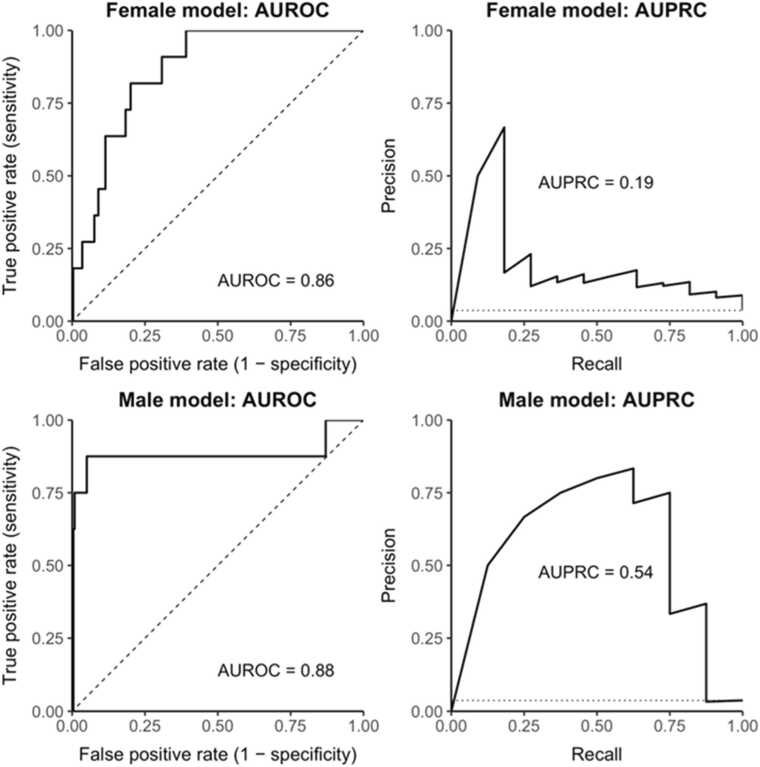
Sex-Specific Prediction Model Performance. *Note*. AUROC =  Area under the receiver operating characteristic curve, AUPRC =  area under the precision-recall curve.

Table 2 shows the top predictors of past 30-day e-cigarette use in sex-stratified models for females and males. For both sexes, cannabis vaping emerged as the strongest predictor. In the female model, a broader set of predictors contributed to model performance. Notably, mental health indicators, including depression and post-traumatic stress disorder (PTSD), were among the most influential predictors for female adolescents, but not for their male counterparts. In contrast, the male model relied heavily on only a few predictors, such as cannabis vaping and cannabis smoking, with other variables contributing substantially less, resulting in a steeper decline in importance values. Given that scaled variable importance was normalized within each model, the absolute values are not directly comparable between the female and male models. The relatively higher scaled importance observed in the female model reflects a more even distribution of predictive contributions across multiple factors, rather than superior overall predictive performance. Prediction performance for the sex-stratified models was moderate, with AUROC values ranging from 0.86 to 0.88 and AUPRC values ranging from 0.19 to 0.54 (see Fig. 1).

**Table 2:** Variable importance of predictors in the sex-stratified models.

	**Female model**		**Male model**	
	**Variable**	**Scaled variable importance**^**a**^	**Variable**	**Scaled variable importance**^**a**^
1	P30D cannabis vaping	1.00	P30D cannabis vaping	1.00
2	Number of friends using e-cigarettes	0.99	P30D cannabis smoking	0.47
3	P30D CBD/hemp use	0.98	Number of friends using e-cigarettes	0.33
4	E-cigarette belief: nicotine buzz	0.76	P30D CBD/hemp use	0.21
5	P30D alcohol use	0.56	Susceptibility: Friend’s e-cigarette offer	0.08
6	Susceptibility: E-cigarette use next year	0.50	Susceptibility: E-cigarette use next year	0.06
7	Depression diagnosis	0.23	Susceptibility: curious about e-cigarette	0.05
8	Susceptibility: Friend’s e-cigarette offer	0.22	P30D cannabis edible use	0.05
9	P30D cannabis edible use	0.22	E-cigarette belief: nicotine buzz	0.03
10	PTSD diagnosis	0.19	E-cigarette belief: appealing flavor	0.01

*Note*. P30D =  past 30-day, CBD =  cannabidiol, PTSD =  post-traumatic stress disorder.

^a^ Variable importance is measured by a predictor’s selection frequency in tree building and its contribution to reducing overall squared error. When a node splits on a feature, its importance is the decrease in squared error from the parent to child nodes, reflecting the reduction in response variance

Table 3 depicts the ranked importance of variables in the combined (female and male) model used to predict adolescent past 30-day e-cigarette use. The most influential predictors primarily consisted of substance use behaviors, including cannabis, alcohol, and combustible tobacco use, as well as factors related to e-cigarette susceptibility, beliefs, and environmental exposure (e.g., the number of friends using e-cigarettes). This pattern is largely consistent with the sex-stratified models and underscores the robust associations between the leading predictors in this model and adolescent e-cigarette use. The directions of associations between these predictors and e-cigarette use are reported in **Appendix Table A.3**. The combined model achieved an AUROC of 0.94 and an AUPRC of 0.54, suggesting that the model demonstrated strong overall predictive accuracy and performance (**Appendix Figure A.1**).

**Table 3:** Variable importance of predictors in the combined (male+female) model.

	**Variable**	**Scaled variable importance**^**a**^
1	P30D cannabis vaping	1.00
2	P30D CBD/hemp use	0.41
3	P30D cannabis smoking	0.36
4	Number of friends using e-cigarettes	0.30
5	P30D alcohol use	0.21
6	Susceptibility: E-cigarette use next year	0.21
7	P30D combustible tobacco use	0.18
*8*	E-cigarette belief: nicotine buzz	0.15
9	Approval of using e-cigarettes	0.10
10	Susceptibility: Friend’s e-cigarette offer	0.08

*Note*. P30D =  past 30-day, CBD =  cannabidiol.

^a^ Variable importance is measured by a predictor’s selection frequency in tree building and its contribution to reducing overall squared error. When a node splits on a feature, its importance is the decrease in squared error from the parent to child nodes, reflecting the reduction in response variance.

## 4 Discussion

The current study was the first behavioral investigation to utilize an ML framework, examining sex differences in risk profiles of adolescent e-cigarette use using comprehensive and contemporary data from Southern California. E-cigarette use prevalence was comparable between females (3.7%) and males (3.5%), and we observed some shared risk factors across sexes, including substance use behaviors, particularly cannabis use, and e-cigarette use susceptibility. However, mental health symptoms, such as depression and PTSD, were identified as prominent risk factors only among female adolescents, underscoring the need for sex-sensitive approaches to screening and prevention programs for adolescent e-cigarette use. In addition, ML models in this study revealed relatively strong predictive performance in the context of behavioral research (e.g., AUROC reaching 0.86–0.88 for sex-stratified models and 0.94 for female+male combined model), despite the highly lopsided nature of the binary outcome, suggesting that ML can provide a promising analytical approach in predicting outcomes with rare cases (e.g., substance use behaviors in adolescence).

Our finding that mental health symptoms, depression, and PTSD emerged as top predictors among female adolescents, not among males, may be explained by differences in the coping strategies associated with stress and emotional challenges across sexes. Compared to males, female adolescents tend to internalize emotional distress and have higher rates of depression, anxiety, and trauma-related symptoms, possibly increasing their reliance on maladaptive coping strategies, such as substance use (Compas et al., 2017, Zahn-Waxler et al., 2008). E-cigarettes might have been used to alleviate negative affect when supportive coping methods are lacking among female adolescents with psychological/emotional distress. It is also possible that some stressors (e.g., body image distortion/dissatisfaction, interpersonal trauma) that are known for elevating risk for both substance use, and mental health symptoms might have disproportionately affected females (Graber, 2004, Johnson et al., 2015). Taken together, our findings highlight that sex-specific strategies for adolescent e-cigarette use prevention (e.g., integrating substance use behaviors and mental health among female adolescents) are needed rather than uniform approaches.

Another key finding of this study was that cannabis use behaviors emerged as the top risk factors for e-cigarette use among both female and male adolescents. Among adolescents, engaging in one type of substance use (e.g., cannabis use) may serve as a proxy for a broader risk-taking behavior in peer networks that normalizes other substance use (e.g., e-cigarette use) (Kowitt et al., 2015). In this study, peer influence (number of friends who use e-cigarettes) was also one of the strong risk factors in both sexes, potentially indicating that those who use cannabis may be situated within peer networks that encourage polysubstance use, including e-cigarettes. In addition, a common liability (e.g., biobehavioral/neurodevelopmental or psychosocial factors) might have elevated the risk of using both cannabis and e-cigarettes (Vanyukov et al., 2012). It is also possible that shared motivations (e.g., mood regulation, stress relief) for using psychoactive substances may contribute to dual use of cannabis and nicotine (Rabin and George, 2015). A systematic review and meta-analysis revealed that adolescents who use e-cigarettes are significantly more likely than those who do not use e-cigarettes to report cannabis use, and this association was stronger among adolescents (vs. young adults, Chadi et al., 2019). Among the different modes of cannabis administration, cannabis vaping was the most influential risk factor of adolescent e-cigarette use. This finding suggests potential heterogeneity in the magnitude of associations across different cannabis product types (Evans-Polce et al., 2025, Wang et al., 2022). Adolescents who vape cannabis may already be familiar with vaping techniques and product features and social contexts, which may in turn facilitate e-cigarette use – or conversely, prior experience in e-cigarette use may increase the likelihood of initiating cannabis vaping. It is also plausible that some adolescents may perceive vaping as more socially acceptable and less harmful than combustible products (e.g., smoking cannabis or cigarettes, Berg et al., 2015), which might have contributed to the increased risk of dual use of e-cigarettes and cannabis vaping.

This study has limitations. While this study leveraged contemporary and flexible data that capture a broad and evolving range of social-behavioral determinants of adolescent health behaviors, our analyses were still limited to the measures available in the data and may have omitted some important predictors of adolescent e-cigarette use (e.g., genetic factors). This study employed GBM as the ML algorithm for developing e-cigarette prediction models. Although GBM is a powerful tool for binary classification that addresses several limitations of conventional logistic regression and provides strong predictive performance in this study, we cannot rule out the possibility that another ML method might provide an improved prediction performance. In addition, GBM does not offer information on the directionality of associations (i.e., whether risk factors are positively or negatively associated with the outcome), however, this study used a complementary statistical approach (logistic regression) to better understand the direction of the associations. Given the cross-sectional design of the study, we were unable to establish the temporal associations between the identified risk factors and adolescent e-cigarette use. Future analyses incorporating follow-up waves are warranted to examine prospective prediction and strengthen causal inference. All data in this study were self-reported – we cannot eliminate potential reporting bias and recall bias. AUPRC was lower in the female model compared to the male model (0.19 vs. 0.54), indicating that class imbalance and prediction quality differed by sex. This discrepancy may be attributable to greater heterogeneity in predictors among females, which could render precision–recall trade-offs less favorable. Missingness for some predictors was over 20%; while multiple imputation was used to mitigate potential bias, some uncertainty in the estimates may remain. Lastly, using more harmful substance use behaviors (e.g., cannabis, tobacco) to predict e-cigarette use may appear counterintuitive. However, this reflects a conceptual rather than methodological concern. In this study, other substance use behaviors serve as indicators of shared vulnerability and clustered risk, situating e-cigarette use within a broader adolescent risk behavior framework rather than implying a hierarchy of harm.

In this ML analysis of adolescents from Southern California, cannabis use behaviors consistently emerged as the top risk factors of e-cigarette use among both females and males. Importantly, we found sex differences in e-cigarette use risk profile – two mental health symptoms, depression and PTSD, were among the most influential risk factors for e-cigarette use among female adolescents, but not for male counterparts. Our findings highlight the importance of tailoring adolescent prevention/intervention programs to account for sex differences, particularly by integrating mental health screening and support into efforts aimed at reducing adolescent e-cigarette use. In addition, prevention efforts targeting substance use and mental health should account for their co-occurrence, particularly among emotionally vulnerable groups such as female adolescents.

## CRediT authorship contribution statement

**Raina D. Pang:** Writing – review & editing. **Jimi Huh:** Writing – review & editing. **Dae-Hee Han:** Writing – original draft, Funding acquisition, Formal analysis, Conceptualization. **Danyi Li:** Writing – original draft. **Adam M. Leventhal:** Writing – review & editing, Funding acquisition. **Ming Li:** Writing – review & editing. **Jessica L. Barrington-Trimis:** Writing – review & editing, Funding acquisition.

## Ethical statement

Written parental consent and student assent were obtained prior to data collection. This study was approved by the University of Southern California Institutional Review Board.

## Role of the funder/sponsor

The funders had no role in the design and conduct of the study; collection, management, analysis, or interpretation of the data; or preparation, review, or approval of the manuscript.

## Funding

Research reported in this publication was supported by the 10.13039/100000038FDA/10.13039/100000054NCI under Award Numbers U54CA180905 and the FDA/NIDA under Award Numbers K99DA058241. Research reported in this publication was supported in part by the Cancer Prevention and Control Research Program of Winship Cancer Institute of Emory University and NCI under award number P30CA138292.

## Declaration of Competing Interest

The authors declare that they have no known competing financial interests or personal relationships that could have appeared to influence the work reported in this paper.

## Data Availability

Data and study materials are available from the authors upon reasonable request.
