# Supplementary material for: Identifying risk profile for adolescent e-cigarette use: A sex-stratified machine learning analysis
Source: Drug Alcohol Depend Rep. 2026 Mar 10;19:100427. doi: 10.1016/j.dadr.2026.100427 (PMC12996931; doi:10.1016/j.dadr.2026.100427)
Supplement: Supplementary file 1 — Supplementary material [file mmc1.docx]

**Appendix Table A.1**. Predictor variables included in the primary analysis

| **Predictors** |
| --- |
| **Sociodemographic characteristics** |
| Age; Sexual identity; Race & ethnicity; BMI, Parent educational attainment, Household dwelling type, School performance (grades) |
| **Past 30-day substance use behaviors** |
| combustible tobacco use; non-combustible tobacco use; marijuana smoking; marijuana vaping; marijuana or THC edibles, foods, or drinks use; any CBD or hemp product use; alcohol use |
| **E-cigarette susceptibility and belief measures** |
| Use in the next year: Any electronic cigarette; Curious: Any electronic cigarette; Approval: Vaping nicotine or using e-cigarettes; More socially acceptable than smoking cigarettes; Provide a good nicotine buzz; Can be used without other people knowing; Less harmful than cigarettes; Easier to hide or conceal than smoking cigarettes; Come in appealing flavors; Help people quit smoking cigarettes; Can be used in places where smoking isn’t allowed; Number of friends who use e-cigarettes; Friend offered: Any electronic cigarette |
| **Exposure to e-cigarette promotion** |
| See posts that promote the use of e-cigarettes and nicotine vaping products; See posts that discourage the use of e-cigarettes and nicotine vaping products; TV/Movie Ads: Vaping |
| **Phone use behaviors** |
| Number of hours you spend on your phone each day on school days; Number of hours you spend on your phone each day on non-school days; Phone behavior: Parents put limits on your screen time; Phone behavior: Parents put limits for when your apps shut down; App used most often |
| **Biggest issues that teens face** |
| Mental Health; Academic stress/time management; Financial stress/poverty; Drugs/substance use; Family relationships; Peer relationships; Plans after high school; Social justice issues; Social media |
| **Perceived neighborhood disorder** |
| Physical disorder; Social disorder |
| **Impulse behavior scale (UPPS-P)** |
| Sensation seeking, Premeditation |
| **Multidimensional Facebook intensity scale** |
| Persistence, Boredom, Over use, Self-expression |
| **Everyday Discrimination Scale** |
| **Parent-adolescent communication scale** |
| **Self-reported psychiatric symptoms** |
| General anxiety disorder, Major depressive disorder, Obsessive compulsive disorder, Jenkins Sleep Scale |
| **Physical and mental health diagnoses made by a health professional** |
| Attention Deficit Disorder (ADHD), Asthma, Diabetes, Cancer, Digestive or gut issues, Depression, Anxiety, Post Traumatic Stress Disorder (PTSD), Autism Spectrum Disorder (ASD) |

**Appendix Table A.2**. Hyperparameter search space and trained values

|  | **Hyperparameter** | **Search space** | **Trained value** |
| --- | --- | --- | --- |
| Combined | col_sample_rate | c(0.05, 0.3, 1.00) | 0.05 |
|  | learn_rate | c(0.001, 0.01, 0.1) | 0.01 |
|  | max_depth | seq(1, 10, by=1) | 1 |
|  | ntrees | c(100, 500, 1000) | 500 |
|  | sample_rate | c(0.05, 0.3, 1.00) | 0.3 |
| Female only | col_sample_rate | - | 0.05 |
|  | learn_rate | - | 0.01 |
|  | max_depth | - | 2 |
|  | ntrees | - | 1000 |
|  | sample_rate | - | 0.05 |
| Male only | col_sample_rate | - | 0.3 |
|  | learn_rate | - | 0.001 |
|  | max_depth | - | 9 |
|  | ntrees | - | 500 |
|  | sample_rate | - | 0.3 |

*Note*. For each model, the area under the receiver operating characteristic curve was used as the optimization metric during tuning and testing. The search space for female-only and male-only models is identical to that of the combined model.

**Appendix Table A.3**. Results from binary logistic regression models

| **Variable** | **OR** | **95% CI** |
| --- | --- | --- |
| P30D cannabis vaping, yes | 90.97 | 46.77, 183.19 |
| P30D CBD/hemp use, yes | 58.53 | 27.76, 127.65 |
| P30D cannabis smoking, yes | 52.45 | 26.84, 104.28 |
| Number of friends using e-cigarettes | 2.43 | 2.12, 2.80 |
| P30D alcohol use, yes | 23.04 | 12.61, 41.68 |
| Susceptibility: E-cigarette use next year | 5.05 | 3.78, 6.80 |
| P30D combustible tobacco use, yes | 62.79 | 21.58, 207.23 |
| E-cigarette belief: nicotine buzz | 4.06 | 3.01, 5.56 |
| Approval of using e-cigarettes | 2.88 | 2.23, 3.78 |
| Susceptibility: Friend’s e-cigarette offer | 4.16 | 3.16, 5.50 |

*Note*. Separate models were estimated for each predictor to avoid instability in estimates due to multicollinearity. OR = odds ratio, CI = confidence interval, P30D = past 30-day, CBD = cannabidiol.

**Appendix Figure A.1**. Prediction model performance in combined-sex analysis


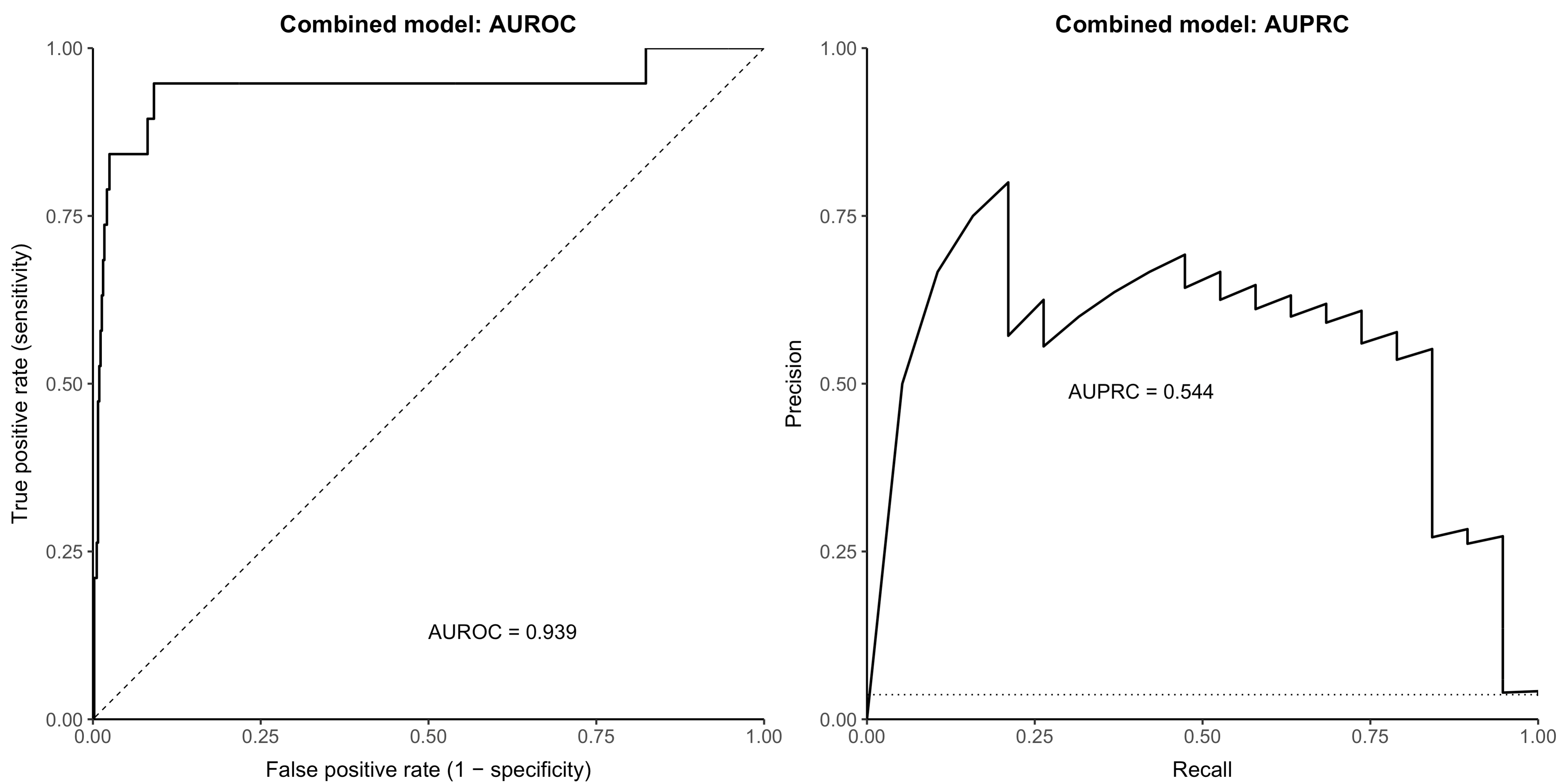


*Note*. AUROC = Area under the receiver operating characteristic curve, AUPRC = area under the precision-recall curve.
